# Microbiological Quality of Pig Carcasses in a Slaughterhouse under Risk-Based Inspection System

**DOI:** 10.3390/foods11243986

**Published:** 2022-12-09

**Authors:** Luciana Giacometti Cavalheiro, Luisa Aneiros Gené, Arlei Coldebella, Jalusa Deon Kich, Vera Letticie de Azevedo Ruiz

**Affiliations:** 1 School of Animal Science and Food Engineering, University of São Paulo, Av. Duque de Caxias Norte, 225, Pirassununga 13635-900, Brazil; 2 Department of Food Science, State University of Campinas, Cidade Universitária Zeferino Vaz, Campinas 13083-970, Brazil; 3 Embrapa Suínos e Aves, BR153, km 110, Concórdia 89715-899, Brazil

**Keywords:** meat production, food safety, risk-based inspection, pork processing, *Salmonella enterica*, mesophilic aerobic counts, *Enterobacteriaceae*

## Abstract

Meat product inspection is one of the procedures adopted more than a century ago to guarantee food quality and safety for consumption. Due to technology and regulation advancement for farming and slaughtering pigs, a change in zoonotic profile attributed to pork has been identified. Thus, a global movement began to establish inspection parameters based on epidemiological risk profiles, culminating in the publication of a new regulation in Brazil in 2018. This normative instruction establishes that slaughterhouses under federal inspection must implement risk-based inspection until 2028. Changes in the inspection system can generate questions and objections on the part of customers and consumer markets. In order to assess microbiological contamination when adopting a risk-based inspection system, the occurrence of *Salmonella* spp. and the quantification of *Enterobacteriaceae* and mesophilic aerobic counts were compared in pig carcasses slaughtered under traditional and risk-based inspection systems. A statistical significance reduction was identified regarding the quantification of *Enterobacteriaceae* (log −0.18 to −1.61 CFU/cm^2^) and mesophilic aerobic counts (log 4.60 to 3.49 CFU/cm^2^). The occurrence of *Salmonella* spp. did not show a significant difference (4% to 5.3%). The results allowed us to conclude that adopting risk-based inspection systems improves food safety through *Enterobacteriaceae* and mesophilic aerobic counts reduction.

## 1. Introduction

Inspection procedures for products of animal origin are adopted in order to ensure the health of consumers by providing them with safe foods [1]. These procedures were first described by the German veterinarian Robert Von Ostertag, with the publication of the *Handbook of Meat Inspection* (Handbuch der Fleischbeschau für Tierärzte, Ärzte und Richter) for veterinarians, doctors, and judges, in 1892. This manual became a world reference and was the model used in the creation of Brazilian legislation by the Ministry of Agriculture, Livestock and Supply (MAPA), such as Ordinance No. 711 of 1995, which advocates inspection procedures in pig slaughterhouses in the country [2,3,4].

Over the years, new technologies and regulations have been introduced into the primary production system, changing the zoonotic profile attributed to pork [5]. Diseases that were common in the past such as tuberculosis, brucellosis, and trichinosis have become rare, while other hazards have increased in relevance, such as *Salmonella* spp., *Campylobacter* spp. and *Yersinia* spp. [6,7,8]. This changes in the zoonotic profiles has rendered the traditional system of sanitary inspection obsolete since it does not allow the identification of the primary microbiological hazards arising from foods. Frequently detected lesions currently have no impact on public health, while the palpation and incision techniques used favor bacterial cross-contamination [6,9,10,11,12]. These findings have been confirmed by modelling, which estimates an increase in *Salmonella enterica* contamination in pig carcass from 1.2% to 95.7% [13].

Studies showed that visual inspection of carcasses and viscera mitigates cross-contamination, which guided new legal requirements in inspection systems in the European Union (EU), Australia, the United States and Canada [6,7,10,13,14,15,16,17,18]. In Europe, there is a variation in the status of meat inspection reforms, and the transition is ongoing, where 61% of the countries have implemented the new system [19].

In this context, inspection system modernization is necessary to improve public health and consumer safety in a cost-effective way, to increase efficiency, optimize resources and reduce waste and losses [1,7,16,20,21,22,23]. Countries that implemented the risk-based inspection reported reduced or equal workloads [19].

Among those countries that implemented the new system, stakeholders (competent authority, personnel, meat inspectors and food business operators) tend to have higher confidence in the new systems than in the traditional system, and believe that it ensures a higher degree of food safety. Nevertheless, the involvement of food business operators’ staff in meat inspection raised concerns, particularly among the relevant authorities, regarding their ability to judge neutrally [19]. Alban [24] has reported that there is a fear of losing their jobs that could negatively influence their perception of the meat safety and public health benefits of changes.

In countries with systemic issues (e.g., local authorities lacking staff and highly fragmented jurisdictions), the confidence that a new system could be implemented successfully was lower. It is to be expected that these countries will face more resistance in implementing the new meat inspection systems [19].

In the European Union, the most common barriers to implement risk-based inspection are trade agreements with third countries, resistance from meat inspectors, inadequate food chain information, and the cost of implementation [5,19,25]. In order to overcome any hurdles, a transparent and gradual shift, focused and careful training, comprehensive and coordinated communication, impact testing and development of individual guidelines focused on food safety using a risk-based approach [5,19,25,26] are recommended.

Thus, in Brazil, in order to prioritize public health hazards through the consumption of pork products, a regulatory modernization of inspection took place based on risk analysis and a review of inspection procedures adopted at the time [16,27]. This movement culminated in the publication of Normative Instruction No. 79 by the Ministry of Agriculture, Livestock and Supply (MAPA), which approved and made mandatory the implementation by 2028 of a new ante-mortem and post-mortem inspection procedures for pigs based on risk in Brazil [28].

The Brazilian Agricultural Research Corporation (Embrapa), together with MAPA, carried out qualitative and quantitative risk assessments involving mapping the history of detections in slaughterhouses and the public health hazards related to products of pig origin, reviewing current legislation, and validating new procedures in pilot units. However, since the change is recent, available research on the topic in Brazil remains scarce [16].

The qualitative risk assessment was restricted to intensive industrial swine production, which considers herds classified as industrial, where there is veterinary control of the animal origin, feed, technical assistance and access to farms. This assessment identified 124 hazards related to unprocessed pork consumption in Brazil and among 19 pathogens considered in the list, only *Salmonella* spp. was classified as high risk [27,29].

Salmonellosis is one of the main public health zoonoses in the world where pork is the associated food vehicle [12]. Most serotypes of *Salmonella enterica* subspecies enterica are pathogenic for humans and can cause food poisoning, severe infections and even death [30]. Pigs, as carriers of *Salmonella enterica*, are crucial for carcass contamination in the production process. The main sources of direct and indirect contamination are their feces, lymph nodes, skin and mouth [31,32].

Another microbiological evaluation used is the *Enterobacteriaceae* and mesophilic aerobic counts as indicators of contamination and hygienic-sanitary conditions in processing [33]. The main pathogens related to *Enterobacteriaceae* are *Escherichia coli*, some serotypes of *Salmonella enterica* and some species of *Yersinia* spp. [34]. A high quantity of *Enterobacteriaceae* indicates a possible presence of relevant pathogens and has a direct correlation with *Salmonella enterica’s* presence in pig carcasses [33].

Concerning mesophilic aerobic counts, a high quantification suggests contaminated raw material, insufficient sanitary food processing or time-temperature abuse, when food remains out of ideal conservation conditions. It is a limited assessment considering that a high count does not represent the presence of pathogens; on the other hand, it indicates that there were favorable conditions for their multiplication [35].

In order to follow the risk assessment guidelines, the new Brazilian legislation establishes that risk-based inspection is exclusively applicable to establishments that slaughter pigs raised in integration and corporativism systems confinement or from independent breeders, duly registered in the official animal health service. Risk-based inspection is not applicable for breeding pigs, other species of suidae or any free-living or field-bred swine at any stage of the production or wild slaughtered by hunting, which may carry endemic pathogens, such as Taenia solium and Toxoplasma gondii [16,28].

Regarding the statement made by the EFSA (European Food Safety Authority) about favoring microbiological cross-contamination by incisions and palpations, the normative instruction provides procedures with fewer incisions, as suggested by Embrapa. However, there are no published studies in Brazil that demonstrate the reduction of microbiological contamination in carcasses due to the lower number of incisions throughout the slaughter process in risk-based inspection systems [6,16,28].

Pork is the leading source of animal protein produced in the world, accounting for 107 million tons in 2021. Brazil is the fourth-largest producing and the fourth-largest exporting country, shipping its products to more than 100 countries [36]. Due to its prominent position in this scenario, changes in the inspection system can generate questions and objections on the part of customers and consumer markets. Therefore, the present survey provides scientific data to support sanitary control programs, reassuring the sector regarding the necessary adjustments in the production chain.

## 2. Materials and Methods

This survey was approved by the Research Ethics Committee of the School of Animal Sciences and Food Engineering of the University of São Paulo (FZEA-USP). It was carried out in November 2020 at a pig slaughterhouse of a federally inspected Brazilian company, which receives pigs from farms under a vertically integrated production system. The daily slaughtered volume is 7500 pigs, running 550 pigs per hour, operating in two production shifts, each one with its own personnel. 

The survey was conducted to evaluate the hygienic-sanitary conditions of carcasses produced under the traditional inspection system versus a risk-based system, comparing the surface contamination of carcasses in both systems.

### 2.1. Adaptation of the Production Process

The current slaughtering process adopted at the facility employs the traditional inspection system, which is based on the procedures described in Decree No. 711 of 1995 [2]. Thus, to perform samplings in this system, there was no need for training or for a process stabilization phase before obtaining the samples, as all teams are trained once a year by the federal inspection service.

In order to adapt to the risk-based inspection system, training was carried out for the employees involved in the ante and post-mortem stages of slaughter based on new procedures and provisional structural adjustments according to the requirements described in Normative Instruction No. 79 of 2018, shown in Table 1 [28]. The structural adjustments did not change any hygienic practices or protocols besides the ones addressed in the normative instruction.

One veterinarian was designated as responsible for all ante-mortem activities and another one for all post-mortem activities. The veterinarians in charge, the processor leaders and the team of researchers received 48 h formal training from EMBRAPA (official regulatory training). After formal training, a test was required to certify the participants.

In order to ensure that procedures were followed as established by regulation, production personnel and inspectors received on-the-job training by the veterinarians in charge, connecting knowledge, purpose and practice. After on-the-job training, no test was required, as all procedures were supervised the whole time by the veterinarians.

Immediately following the exsanguination process, all carcasses were dehaired through scalding (62–72 °C for 2 to 5 min), scraping (automatic dehairing), buffing, singeing, and were washed with water (3 atm pressure). During the dressing process the carcasses were split in two sides and, before entering the cooler, they were washed one more time.

Regardless of inspection systems, the production plan is shared with the official inspection service and every product destined for human consumption is registered with an official inspection service platform and communicated to the whole team [3].

The risk-based system was adopted in the first shift over three weeks, the first two weeks for process stabilization and procedure adjustments, and the last week for conducting sample collection. The first shift was active from 5:00 a.m. to 3:00 p.m., considering that the slaughter activity started at 5:00 a.m. and finished at 2:00 p.m. 

For six weeks, three weeks for each system, the official inspector, processor and the team of researchers were present to make sure all procedures were being well executed and would not influence one system more than the other.

### 2.2. Sampling Plan

A randomized block design was used in the survey to evaluate two treatments: (1) a traditional inspection system; and (2) a risk-based inspection system, with blocks at the collection times throughout the shift to identify the influence of time on each inspection system and possible interactions between both factors.

Samplings were carried out in the first shift for five days on carcasses inspected under the traditional system and five days under the risk-based system, always at the following times: 5:30 a.m., 8:30 a.m., 11:30 a.m., and 2:30 p.m. At each time and date, samples of five carcasses were collected, totaling 20 carcasses per day per inspection system and 200 carcasses throughout the survey. 

Each sample was sent to an external laboratory for total *Enterobacteriaceae* and mesophilic aerobic counts, given that they are the leading indicators of hygienic-sanitary conditions in the pig slaughter process, and to investigate the presence of *Salmonella enterica*, due to its characterization as a high-risk pathogen, for a total of 600 laboratory analyses [29].

The samplings were carried out for five days in each inspection system to increase the accuracy of the data by minimizing the influence of the day and other uncontrolled factors that could influence the obtained results.

The sampling plan was based on the guidelines of the International Commission on Microbiological Specifications for Foods [37], which defines the number of units to be collected (n = 5) per evaluated batch.

### 2.3. Sample Collection and Analysis Procedure

**Figure 1:**
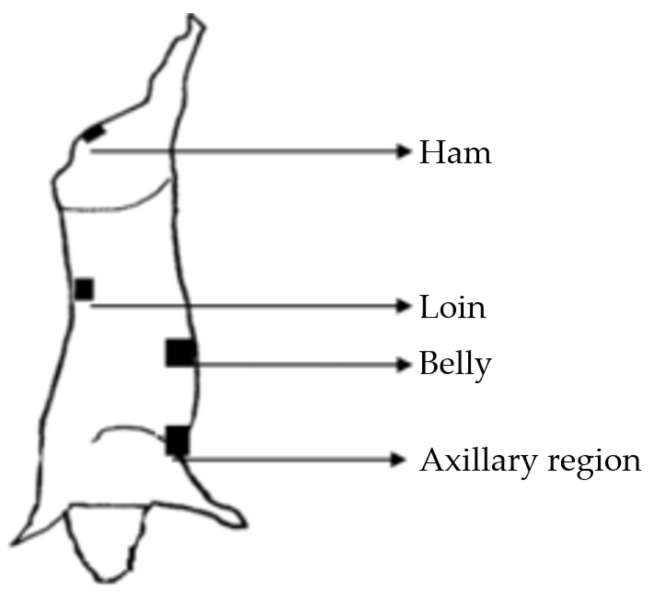
Pig carcass sampling sites.

The sampling procedure was carried out based on Normative Instruction No. 60, which establishes microbiological control in swine carcass, in bovine carcass and meat in slaughterhouses in Brazil [38]. One side of the carcass was chosen randomly, and its skin was swabbed using a sterile sponge (3M), covering four points of each carcass (ham, belly, loin, and axillary region), totaling 400 cm^2^ (Figure 1). The step-by-step procedure followed the provisions as required by Brazilian Ministry of Agriculture, Livestock and Supply, a similar method to that recommended by the European Union [39,40].

The swabbing of the carcass surface was conducted before entering the cooling system, right after its final wash and before any intervention. This is in attempt to reduce the biological risk, by means of friction, using a sterile cellulose sponge that was pre-hydrated in biocide-free buffered peptone water. The person responsible for sample collection sanitized their hands with 70% alcohol and wore sterile gloves and a mask. A 100 cm^2^ square sterile template was used to guide the sampling site, starting at the ham, followed by the loin with one side of the sponge, and the other side of the sponge was used on the belly and axillary region. Sampling was performed with ten horizontal and ten vertical movements for each area.

At the end of the sample collection, each sponge, which represented 400 cm^2^ of a carcass, was stored in an individual sealed sterile plastic bag that was properly identified (date, time, shift, and batch) and kept under refrigeration at a temperature of 1 °C to 8 °C until processing in an outsourced MAPA-accredited laboratory.

Each sample was evaluated under a *Salmonella* spp. detection method (AFNOR) and a quantification of total *Enterobacteriaceae* and mesophilic aerobic counts was carried out under the Dry Rehydratable Film Method, Petrifilm™ *Enterobacteriaceae* Count Plate (AFNOR) and Petrifilm™ Aerobic Count Plate (AOAC) [41,42,43]. In cases where *Salmonella* spp. results were positive, the samples were also evaluated also under the ISO method for confirmation [44].

Briefly, in the *Salmonella* spp. detection method, buffered peptone water supplemented with a *Salmonella* spp. colored supplement is added to the moistened sponge (1:10) and incubated at 41.5 °C for 18 h. The sample is boiled for 5 min at 95 °C and 0.5 mL is used to perform a VIDAS^®^ test [41].

In the Dry Rehydratable Film Method, buffered peptone water is added to the moistened sponge to achieve the total volume of 100 mL and homogenized. The Petrifilm is placed on a level surface and the top film is lifted. Using a pipettor, 1 mL of diluted sample is placed onto the center of the bottom film and the top film is dropped down onto the sample. The Petrifilm is incubated at 37 °C for 24 h for the *Enterobacteriaceae* Count Plate and at 35 °C for 48 h for the Aerobic Count Plate [42,43].

In this study, as each sponge sample is diluted to 100 mL and the procedure follows the equivalence of mL/cm^2^, each sample of 100 mL is equivalent to 100 cm^2^. According to ISO [45]:

Ns = ((N × F)/A) × D

Considering N the number of cfu in 1 mL of dilution liquid; F the amount of dilution fluid in tube or homogenizer bag, in milliliters; A the sampled surface, e.g., in square centimeters and D the reciprocal of the dilution used. In cases which the test sample contains no colonies the result must be reported as “less than 1/d ufc/mL” where d is the dilution factor of the initial suspension. In this approach, the minimum limit of 1 must be divided by 4, as 4 is the dilution factor, which implies to:

Ns = ((1 × 100)/400) × 1 = 0.25 cfu/cm^2^

All samples were registered in a test code, not composing or interfering with the slaughterhouse collection cycles, according to Normative Instruction No. 60/2018 [38].

If the obtained results were above the maximum acceptable values established in the current legal requirements for *Salmonella enterica* and *Enterobacteriaceae* in force, the slaughterhouse would be required to identify the cause and take corrective and preventive actions to reestablish process control [38].

### 2.4. Statistical Analysis

The statistical analysis for the presence of *Salmonella enterica* in the collected samples was performed using Fisher’s exact test to compare the two inspection systems using the FREQ procedure of the SAS software [46].

For *Enterobacteriaceae* and mesophilic aerobic counts, results were obtained in CFU/cm^2^ and were fitted using the maximum likelihood method and lognormal distribution for censored data with PROC LIFEREG [46], due to restrictions in the counting methodologies. The method is suitable for estimating distributions with censored data, as is the case for CFU results and microbiological testing. 

In this approach, a negative result is considered left censored (i.e., less than one bacterium in 4 cm^2^ or less than 0.25 CFU/cm^2^). Positive samples and in a finite number of CFU are considered uncensored data. Finally, positive samples and with infinite number of CFU (i.e., >250 CFU/cm^2^) are considered right censored data. The effects of treatment, sampling time and the interaction between treatment and sampling time were evaluated in the analyzed model. When a significant (*p* ≤ 0.05) effect of sampling time or interaction was detected, a Tukey’s test was performed.

The means of each inspection system were calculated from the logarithmic results of each sample (log (CFU/cm^2^). All samples results were considered. A Wald Chi Square test was performed to compare both inspection systems.

The following factors were controlled and stable: sampling shift, hygiene procedures, the person responsible for sample collection, handlers, and the genetics and management on the farm of origin of the animals; they were not considered as fixed factors in the survey.

## 3. Results

At the end of the two weeks of sample collection, with an interval of two weeks between the two for process adaptation and stabilization, 195 samples had been collected, and 585 microbiological analyses had been performed. One hundred ninety-five analyses for each microorganism were conducted: the investigation for *Salmonella enterica*, the quantification of *Enterobacteriaceae*, and the quantification of mesophilic aerobic counts.

In total, 100 samples were collected in the traditional system, accounting for all days and times proposed. Meanwhile, in the risk-based system, 95 samples were collected due to the lack of sample collection in the first hour of the second risk-based inspection system day on account of a lack of sampling materials.

### 3.1. Frequency of Salmonella enterica Occurrence

In order to investigate for *Salmonella* spp., a total of 195 analyses were performed, nine of which (4.61%) were positive. When comparing the two inspection systems, a frequency of 4% was observed in the traditional system, while in the risk-based system, the frequency was 5.3% (Table 2).

The nine positive samples occurred on different days and times, showing no relationship with the inspection systems evaluated.

No significant difference was observed in the frequency of *Salmonella* spp. isolation between the two systems (*p* = 0.7427). Due to the low frequency of *Salmonella enterica*, a statistical analysis was not performed in the evaluation between collection days and times.

### 3.2. Quantification of Enterobacteriaceae and Mesophilic Aerobic Counts

The quantification of bacteria of the Family *Enterobacteriaceae* resulted in a mean of −0.18 log (CFU/cm^2^) for the traditional inspection system and −1.61 log (CFU/cm^2^) for the risk-based inspection system. As for the mesophilic aerobic counts, the results implied a mean of 4.60 log (CFU/cm^2^) and 3.49 log (CFU/cm^2^), respectively.

Considering both the *Enterobacteriaceae* and the mesophilic aerobic counts, lower mean counts were observed in the risk-based inspection system compared to the traditional inspection system. When performing statistical analysis using the Wald Chi Square test, a significant difference was found in both cases (*p* < 0.0001) for the principal effect of the inspection system (Table 3).

Table 3 also shows the comparisons of the sample collection times for the *Enterobacteriaceae* and mesophilic aerobic counts in each employed inspection system, which reveals that the interaction was significant for Treatment X Sampling Time effect to *Enterobacteriaceae* count.

It can be noted that the collection time only affected the *Enterobacteriaceae* count in the risk-based inspection system and for the mean of treatments, with the lowest count occurring at 5:30 a.m. In the traditional inspection system, there were no significant differences (*p* > 0.05) regarding the collection times. At 5:30 a.m. and 2:30 p.m., a risk-based inspection system had less count of *Enterobacteriaceae* then the traditional inspection system, however at 8:30 a.m. and 11:30 a.m. there was no difference between treatments. Mesophilic aerobic counts were affected by the systems at 5:30 a.m. and 2:30 p.m. (*p* < 0.05) and were not affected by interaction with the sampling time (*p* > 0.05).

It was verified that almost 50% of the samples collected in the risk-based inspection system presented *Enterobacteriaceae* count results below the detection limit (<0.25 CFU/cm^2^), whereas this percentage was 34% in the traditional inspection system (Table 4).

Meanwhile, in the mesophilic aerobic counts analysis, 33% of the samples in the traditional inspection system obtained higher values than the maximum count identified by the applied methodology (>250 CFU/cm^2^). In contrast, none of the samples in the risk-based inspection system achieved this result (Table 4).

When evaluating the distribution of the results of the *Enterobacteriaceae* and mesophilic aerobic counts per inspection system and collection time, it was noted that only at 5:30 a.m, were more than 50% of the results found in the range <0.25 CFU/cm^2^ (80%) in the risk-based system, a fact that corroborates the statistical difference observed in Table 3.

## 4. Discussion

The complete elimination of risks in food production and consumption is an unrealistic goal. Therefore, risk mitigation analysis is essential for both consumer protection and business sustainability. The microbiological analyses conducted throughout the process are considered key elements in guaranteeing food safety [47].

The intensification of pig production, increased productivity, the adoption of technologies, advances in the sanitary control of herds, and the evolution of the inspection system to mitigate high-risk pathogens has led to the review of inspection and microbiological monitoring procedures, culminating in the updating of legal requirements related to the issue in several countries [6,14,16,17,18,29,48,49].

In Brazil, the publication of a normative instruction that establishes a reduction in the handling and incision of carcasses and viscera may raise questions by those involved in the process, as these procedures have been adopted for decades to detect irregularities.

The proposed inspection system is disruptive and, in a way, educational since it makes it imperative that official inspectors and slaughterhouse employees study the subject and adapt to a mentality focused on the level of risk, which, in most cases, is invisible to the naked eye.

In the case of the present survey, in addition to demonstrating that food can be obtained at levels considered safe in both inspection systems in force in Brazil, it can be noted that the implementation of a risk-based inspection system does not represent a compromise to public health. In fact, when slaughtered pig carcasses were evaluated in this inspection system, the obtained results showed lower levels of microbiological contamination with *Enterobacteriaceae* and mesophilic aerobic counts when compared to the traditional inspection system, even though both were within acceptable levels.

The findings herein corroborate studies carried out in other countries. Based on North American experience, the adoption of risk-based inspection systems is unlikely to increase the occurrence of *Salmonella* spp. It may even result in its reduction, leading to fewer people being infected. In a study conducted by the Food Safety and Inspection Service (FSIS), a decrease from 3.05 to 0.65 was observed when evaluating post-cooling carcasses after the adoption of a risk-based inspection system [17].

In Europe and Australia, the palpation and incision of carcasses and viscera were eliminated in inspection procedures since they represent a means of cross-contamination [15,48]. In Brazil, a stochastic model to quantitatively describe cross-contamination was developed and showed that the prevalence of contaminated carcass surfaces increased from 1.2% to 95.7% due to the cross-contamination during traditional inspection [13]. In Belgium, inspection practices, such as incising the head and tonsils, were associated with higher levels of hygiene indicator bacteria and *Salmonella* spp. [9].

The observed results were expected, given the establishment of legal guidelines in several other countries. Still, this assessment in a Brazilian slaughterhouse was essential since no comparative microbiological studies between traditional and risk-based inspection systems were identified in the literature.

Due to the importance of the issue to public health, there are several published studies evaluating the presence of *Salmonella* spp. in pig carcasses in slaughterhouses that use traditional inspection systems [29,50,51,52,53,54]. Paim et al. [55] detected an 8% frequency of *Salmonella* spp. in a slaughterhouse in southern Brazil, while Kich et al. [56] reported a frequency of 24%, Cê [57] 1.11%, and Corbellini et al. [33] 11.5% in the same region. Outside Brazil, Swanenburg et al. [58] detected a frequency of 8% in the Netherlands, Piras et al. [59] 15.9% in Italy, Baptista [60] 3.7% in Denmark, and Zhou [54] 15% in China.

What can be seen when analyzing these reports is the significant variation in the occurrence of *Salmonella* spp., which ranged from 1.11% to 24%, a fact that may be related to the distinct detection methods, samples used, and different programs and processes applied for sanitary control in slaughterhouses around the world [50].

The result obtained in official sample collections conducted by MAPA in all the federally inspected pig slaughterhouses in Brazil was 6.2% of *Salmonella enterica* occurrence in 2019, according to the guidelines of Normative Instruction No. 60 for microbiological control in cold storage slaughterhouses [38,61]. The official results reported in the European Union from 2015 to 2019 showed a prevalence of 3.88% [62], and in the United States, a prevalence of 2.7% from 2010 to 2011 [63].

In the present survey, the obtained result (4.61%) was lower than those described in other slaughterhouses in the country and within the range of results stated by other authors.

It is important to emphasize that the three main importing countries of Brazilian pork (China, Hong Kong, and Chile) establish the same requirements as Normative Instruction No. 60 as process hygiene criteria for cargo release, with the maximum acceptable limit for *Salmonella* spp. of six positive samples in 40 samples analyzed [38,64,65,66].

According to our survey, a greater positivity for *Salmonella enterica* was observed on the second day of sampling in the risk-based system. It coincided with the report of a decalibration in the evisceration equipment contaminating the carcasses, which was followed by immediate correction. With this fact in mind, it is possible to infer that the result of 5.3% in the risk-based system could have been lower, reaching 2.10%, showing that an even greater level of food safety can be obtained with this inspection system.

As for the analyses of *Enterobacteriaceae* and mesophilic aerobic counts, the availability of studies in the literature is scarcer, which may be related to the lower risk to public health and in this study were evaluated as hygiene and cross-contamination indicators [16,27,67]. In Brazil, Corbellini et al. [33] evaluated 1150 carcasses in thirteen slaughterhouses, and the obtained results regarding the mean quantification of *Enterobacteriaceae* varied between 0.3 and 1.52 log CFU/cm^2^. Cê [57] analyzed 90 carcasses after final washing and obtained a mean of 1.58 log CFU/cm^2^ for *Enterobacteriaceae* and 3.92 log CFU/cm^2^ for mesophilic aerobic counts. Meanwhile, Paim [55] found results that ranged from −0.8 to 0.46 log CFU/cm^2^ for *Enterobacteriaceae* and 1.43 to 2.48 log CFU/cm^2^ for mesophilic aerobic counts.

In other countries, Zweifel et al. [68] obtained results between 0.08 and 0.42 log CFU/cm^2^ for *Enterobacteriaceae* and 2.2 and 3.7 log CFU/cm^2^ for mesophilic aerobic counts in Switzerland. In Italy, Piras et al. [59] found results that ranged from 2.17 to 5.34 log CFU/cm^2^ and 4.69 to 6.35 log CFU/cm^2^, respectively, while, in Sweden, Lindblad [69] obtained results varying from 0.5 to 1.3 log CFU/cm^2^ and 3.4 to 4.6 log CFU/cm^2^, respectively. According to official data from the United States, the result obtained for *Enterobacteriaceae* was 0.76 log CFU/cm^2^, and for mesophilic aerobic counts, 2.02 log CFU/cm^2^ [63].

Inferior results regarding these groups of microorganisms were observed in the present survey, demonstrating the high levels of sanitary hygiene in the establishment assessed compared to others evaluated by other authors, both in the traditional inspection system and in the risk-based one.

Several studies associate hygiene and process indicators such as *Enterobacteriaceae* and mesophilic aerobic counts to *Salmonella* spp. prevalence [9,33,70,71,72]. These publications aim to support the assumption that adherence to Good Manufacturing Practices and Good Hygiene Practices result in a reduction in public health risks [9].

In Brazil, the only microorganism characterized as high-risk related to pork consumption was *Salmonella* spp. [16]. As it is a pathogen of enteric origin, fecal and hygiene indicators fulfill many of the criteria set for ideal food safety indicators for sharing the same habitat and being detected when the pathogen is present in food [71].

A significant reduction was observed in both the *Enterobacteriaceae* and mesophilic aerobic counts in the risk-based inspection system because of the decrease in handling carcasses and viscera by palpation and incision, mainly due to the non-exposure of lymph nodes and the complete removal of the head. The complete removal of the head is a new procedure established by the risk-based inspection regulation. According to these findings and the association between *Salmonella* spp. as a pathogen, and *Enterobacteriaceae* and mesophilic aerobic counts, it is possible to infer an improvement in food safety as a consequence of the reduced level of these hygiene and contamination indicators.

It is noteworthy that all other factors were controlled, such as hygiene procedures, handlers, as well as genetics and management in the farm of origin of the animals. 

Furthermore, it is possible to infer that the difference between both inspection systems regarding mesophilic aerobic counts could have been greater since the maximum detection value of the analysis methodology was 250 CFU/cm^2^. In other words, counts greater than 250 CFU/cm^2^ were recorded as >250 CFU/cm^2^. This difference between systems may be more significant since, in the traditional inspection system, 33% of the results were above the maximum detection limit, whereas in the risk-based inspection system, none of the samples reached that value. Besides that, no other changes were noticed that could affect this survey result.

In both analyses (*Enterobacteriaceae* and mesophilic aerobic counts), the results obtained from the total logarithmic means per inspection system were within the maximum acceptable limits established by Normative Instruction No. 60 and Circular Letter No. 130 [38,73]. While no deviations were detected, no corrective and preventive actions were necessary that could influence inspectors and processors behavior during the survey.

For reference, the level of quantification of *Enterobacteriaceae* in pig carcasses in Brazil is considered acceptable up to 2 log CFU/cm^2^ of the daily average, intermediate up to 3 log CFU/cm^2^, and, when above, unacceptable [38]. For mesophilic aerobic counts, the reference is considered acceptable up to 5 × 10^3^ CFU/cm^2^ of the daily average, intermediate up to 105 CFU/cm^2^, and when above, unacceptable. This criterion is only applicable for exports to the European Union [73].

As for *Salmonella* spp., the three largest importing countries of Brazilian pork establish the same criteria as Normative Instruction No. 60 for *Enterobacteriaceae*, whereas for mesophilic aerobic counts, there is no restriction, except for exports to the European Union [38,64,65,66,73].

The legally acceptable limits for pork production in the European Union are similar to those in Brazil: for *Enterobacteriaceae*—acceptable up to 2 log CFU/cm^2^ of the daily average, intermediate up to 3 log CFU/cm^2^, and when above, unacceptable; for mesophilic aerobic counts—acceptable up to 4 log CFU/cm^2^ of the daily average, intermediate up to 5 log CFU/cm^2^, and when above, unacceptable, and for *Salmonella* spp., up to five positive samples out of 50 samples analyzed [68]. On the other hand, in the United States, there is no legal requirement for the evaluation of pig carcasses in slaughterhouses [74].

When evaluating the influence of the sample collection time throughout the shift, a difference was identified only for the 5:30 a.m. time in the risk-based system regarding *Enterobacteriaceae*. Although it was not possible to determine the cause of this difference, we found that the result was a consequence of the higher proportion of results <0.25 CFU/cm^2^, approximately 80%. In no other time or system was the proportion of results in this range greater than 50% of the obtained results.

Training personnel is considered crucial and essential to improving conditions in slaughterhouses and to reduce bacterial contamination, and is seen as one strategy by which food safety can be increased [75,76,77,78]. In Europe and Brazil, professional training is a food law requirement [28,76].

The relatively low educational level of the workers requires adapted professional training with a strong connection between knowledge and practice, repetitions and informal on-the-job training [76]. During this study, there was no evidence of bias related to the training program, as in both inspection systems the personnel were trained, and their procedures were supervised by certified veterinarians.

The results obtained herein are relevant to the entire pork production chain, as they support the slaughterhouses that have adopted this new inspection system, demonstrating the greater microbiological quality of the pig carcasses and bringing more reliability to markets and consumers regarding the issue of food safety.

This survey presented limitations regarding the minimum and maximum detectable limits for *Enterobacteriaceae* and mesophilic aerobic counts. Thus, we recommend that, in future studies, researchers conduct as many dilutions as necessary to obtain more accurate results. In addition, specifically for scientific research purposes, we suggest that analyses that identify the serovar of *Salmonella* spp. be carried out when samples are positive to enrich the discussion by comparing the findings with those reported by other authors.

Another suggestion would be a broader analysis of the results concerning *Salmonella* spp. and *Enterobacteriaceae* notified to MAPA from all the Brazilian slaughterhouses before and after implementing the risk-based inspection system. This will indeed provide the actual scenario of the country after the adoption of the new inspection system.

## 5. Conclusions

The risk-based inspection system reduces *Enterobacteriaceae* and mesophilic aerobic counts when compared to the traditional inspection system in this specific establishment. It can be suggested that these results reflect the reduction in carcass handling, less exposure of contaminated tissues due to the complete removal of the head, and the suppression of cuts in the carcass and head lymph nodes. Regarding *Salmonella* spp., no differences were found between the inspection systems, both of which rendered results within the accepted limits. Both traditional and risk-based inspection systems presented all results within national regulation requirements, guaranteeing food safety.

The new inspection system implementation is ongoing in many countries, where barriers, hurdles and resistance were reported. Generalizing the observed differences in this survey, it demonstrates that risk-based inspection improves the microbiological quality of pig carcasses by cross-contamination reduction, consequently increasing food safety. These findings serve to reassure the sector when it concerns the implementation of risk-based inspection and its consequences to public health. Future studies using similar analysis methods are indicated after the official implementation of this new inspection system.

## Figures and Tables

**Table 1:** Post-mortem procedures of traditional and risk-based inspection.

Traditional		Risk-Based	
Inspection Line	Procedure	Evaluation and Classification	Procedure
A1—Head and jowl	Visual and incision	Head, jowl, and tongue	Visual
A—Uterus	Visual and palpation	Intestines, stomach, spleen, pancreas, bladder, and uterus *	Visual
B—Intestines, stomach, spleen, pancreas, and bladder	Visual, palpation, and incision		
		Inspection of mesenteric lymph nodes	Visual and incision
C—Heart and tongue	Visual, palpation, and incision	Heart	Visual and incision
D—Lungs and liver	Visual, palpation, and incision	Lungs and liver	Visual and palpation
E—Carcass	Visual and incision of lymph nodes	Carcass	Visual
F—Kidneys	Visual, palpation, and incision	Kidneys *	Visual
G—Brain *	Visual	Brain *	Visual

* The spleen, pancreas, bladder, uterus, kidneys, and brain undergo specific procedures only when intended for human consumption; this is not applicable for the assessment in question [2,29].

**Table 2:** Presence of *Salmonella enterica* distributed per day and time.

Traditional System						Risk-Based System				
Time	Day 1	Day 2	Day 3	Day 4	Day 5	Day 1	Day 2	Day 3	Day 4	Day 5
5:30 a.m.	0	0	1	0	0	0	-	0	0	0
8:30 a.m.	0	0	0	0	0	0	0	0	1	0
11:30 a.m.	0	1	0	0	0	0	3	0	0	0
2:30 p.m.	0	1	1	0	0	0	0	0	0	1
Total	0	2	2	0	0	0	3	0	1	1

**Table 3:** Means and standard deviations of the *Enterobacteriaceae* and mesophilic aerobic counts on the surface of the pig carcasses in function of the employed inspection system and the time of sample collection.

Treatment	Sampling Time				Means	Pr > χ^2^
	5:30 a.m.	8:30 a.m.	11:30 a.m.	2:30 p.m.		
	*Enterobacteriaceae*—log (CFU/cm^2^)					
Risk-Based	−3.293 ± 0.695 ^b^	−0.592 ± 0.466 ^a^	−1.621 ± 0.492 ^ab^	−0.921 ± 0.473 ^ab^	−1.607 ± 0.279	0.0079
Traditional	0.247 ± 0.457	−0.697 ± 0.471	−0.866 ± 0.472	0.593 ± 0.451	−0.181 ± 0.235	0.0666
Mean	−1.523 ± 0.420 ^b^	−0.645 ± 0.333 ^ab^	−1.244 ± 0.344 ^ab^	−0.164 ± 0.328 ^a^		0.0319
Pr > χ^2^	<0.0001	0.8735	0.2640	0.0200	<0.0001	
	Mesophilic aerobic counts—log (CFU/cm^2^)					
Risk-Based	3.613 ± 0.353	3.509 ± 0.316	3.410 ± 0.316	3.449 ± 0.316	3.495 ± 0.163	0.9765
Traditional	4.599 ± 0.330	4.352 ± 0.327	4.296 ± 0.325	5.155 ± 0.347	4.601 ± 0.168	0.2579
Mean	4.106 ± 0.242	3.930 ± 0.227	3.853 ± 0.227	4.302 ± 0.234		0.5234
Pr > χ^2^	0.0414	0.0639	0.0505	0.0003	<0.0001	

^a, b, ab^ Means followed by different letters in the rows differ according to the Tukey’s test (*p* ≤ 0.05).

**Table 4:** Distribution of the results of the *Enterobacteriaceae* and mesophilic aerobic counts in CFU/cm^2^.

Enterobacteriaceae			Mesophilic Aerobic Counts	
CFU/cm^2^	Traditional	Risk-Based	Traditional	Risk-Based
<0.25	34%	49.5%	0%	1.1%
Between 0.25 and 250	66%	50.5%	67%	98.9%
>250	0%	0%	33%	0%
Total	100%	100%	100%	100%

## Data Availability

The data presented in this study are available on request from the corresponding author.
